# Xeno-Estrogenic Pesticides and the Risk of Related Human Cancers

**DOI:** 10.3390/jox12040024

**Published:** 2022-11-21

**Authors:** Vivek Kumar, Chandra Shekhar Yadav, Basu Dev Banerjee

**Affiliations:** 1 Department of Biotechnology, IMS Engineering College, Dr. A.P.J. Abdul Kalam Technical University, Lucknow 226031, India; 2 School of Forensic Science, National Forensic Sciences University, Gandhinagar 382010, India; 3 Environmental Biochemistry & Molecular Biology Laboratory, Department of Biochemistry, University College of Medical Sciences & GTB Hospital, University of Delhi, Delhi 110095, India; 4 Department of Medical Elementology & Toxicology, School of Chemical & Life Sciences, Hamdard University, New Delhi 110062, India

**Keywords:** endocrine disruption, persistence, xenobiotics, disorders

## Abstract

In recent decades, “environmental xenobiotic-mediated endocrine disruption”, especially by xeno-estrogens, has gained a lot of interest from toxicologists and environmental researchers. These estrogen-mimicking chemicals are known to cause various human disorders. Pesticides are the most heavily used harmful xenobiotic chemicals around the world. The estrogen-mimicking potential of the most widely used organochlorine pesticides is well established. However, their effect is not as clearly understood among the plethora of effects these persistent xenobiotics are known to pose on our physiological system. Estrogens are one of the principal risk modifiers of various disorders, including cancer, not only in women but in men as well. Despite the ban on these xenobiotics in some parts of the world, humans are still at apparent risk of exposure to these harmful chemicals as they are still widely persistent and likely to stay in our environment for a long time owing to their high chemical stability. The present work intends to understand how these harmful chemicals may affect the risk of the development of estrogen-mediated human cancer.

## 1. Introduction

Xenobiotics (xeno: foreign; biotic: life form) are chemicals of synthetic origin that are foreign to living systems. These chemicals are not part of the normal metabolic activity of living organisms and therefore may interfere with the functioning of the physiological system. These unwanted chemicals inside the body are known to have harmful effects on functioning and are responsible for diseases in humans. One such detrimental effect caused by some of these chemicals is xeno-estrogenicity [1].

‘Xeno-estrogenicity’ (xeno: foreign; estrogenicity: resembling natural estrogens) denotes the property of foreign chemicals being able to produce responses in physiological systems comparable to estrogens produced by the body by different mechanisms. Xeno-estrogens are a group of chemicals that disrupt the endocrine system by mimicking the activity of natural estrogens or producing a similar response in the body by other mechanisms. Majorly known persistent organochlorine pesticides (OCPs) and other classes of chemicals present in the environment are known to bind estrogen receptors and produce a physiological response equivalent to the hormone in various organs and tissues [2]. Therefore, these chemicals can produce adverse effects such as increasing the risk of estrogen-mediated cancers [3]. These chemicals can easily enter the human body due to their physiochemical characteristics. They not only widely persist in our environment, including water, soil, and food, but also exhibit high volatility at environmental temperatures [4]. Moreover, a much greater risk is rendered by such chemicals due to their biomagnification through to the top of the food chain to humans. Additionally, their lipophilic nature makes them more harmful, as these compounds easily bioaccumulate in the adipose tissue of humans [5]. Therefore, a large portion of the world’s population, if not all, is apparently exposed to these chemicals. The health concerns due to these endocrine-disrupting chemicals are inevitable as exposure to these chemicals is continuous throughout the lifetime [6,7,8]. Studies have shown evidence of the continuous exposure to these compounds as their metabolites have been detected in different types of human samples. Studies reported a statistically significant correlation between cancer risk in individuals and their adipose tissue concentration [9,10].

Xenobiotics, including from pesticide exposure, are significant causes of cancer incidence worldwide [11,12]. These compounds are known to increase the risk of various human cancers amongst humans by different mechanisms such as DNA mutations, inflammation, upregulation of tumor suppressor genes, and/or downregulation of oncogenes [13]. Amongst the harmful effects of these contaminants, xeno-estrogenicity remains one of the most enigmatic and widely reported ones to contribute towards higher morbidity and mortality [14]. These estrogen-mimicking chemicals are known to increase the transcriptional activity factors associated with the natural estrogens, thus enhancing the risk of various estrogen-mediated human disorders including cancer [15,16,17,18].

Other natural and man-made compounds are known to exist with xeno-estrogenic potential in the environment, such as butylated hydroxyanisole, phthalate esters, nonylphenol, benzyl butyl phthalate, and bisphenol-A, etc. [19]. However, pesticides are the most potent xeno-estrogens amongst all the chemicals because the estrogenic potency and half-life in the environment of these compounds exceed those of any other compound [3,4,5].

## 2. Carcinogenic Effects of Organochlorine Pesticides

Pesticides are the most well-known carcinogenic chemicals to humans. Studies from both humans and animals have unambiguously confirmed the development of pesticide exposure-mediated human cancer [20]. All known OCPs possessing xeno-estrogenic potential are listed in the ‘first dirty dozen’ in the Stockholm Convention as they were widely reported to cause various adverse health outcomes in humans around the world [21]. Human studies support the association of various OCPs (aldrin, dieldrin, endosulfan, HCH, DDT, 2,4,5-trichlorophenoxyacetic acid, phenoxy acid herbicides, and methoxychlor). The exposure (both occupational and non-occupational) has been strongly linked to increased incidence of non-Hodgkin’s lymphoma [22]; multiple myeloma [23]; soft tissue sarcomas [24]; lung cancers [25]; and cancers of the pancreas, stomach, liver, and kidney as well as urinary and gall bladder cancer [26,27,28,29,30].

The International Agency for Research on Cancer (IARC) has listed OCPs as potent carcinogens in animal studies [31,32]. Cancer development also depends on genetic susceptibility as well. Xenobiotic metabolic enzymes are metabolic susceptibility enzymes whose metabolization leads to the elimination of a diverse range of xenobiotics, including OCPs. These predominantly hepatic enzymes are responsible for xenobiotic detoxification in two phases: ‘phase I’ enzymes (functionalization reactions), mainly cytochrome P450, and ‘phase II’ enzymes (conjugation reactions), including glutathione S transferases (GSTs). Cytochrome P450 (abbreviated as CYP P450 and infrequently as CYP450) is a superfamily of monooxygenase enzymes that catalyze metabolism by oxidation of their substrates. On the other hand, GSTs, important phase II enzymes of the xenobiotic metabolizing enzyme family, eliminate phase I CYP450 enzymes’ catalyzed intermediates, leading to their excretion out of the body [33,34].

Individual susceptibility to environmental chemical exposure leading to diseases, including cancer, is modified by genetic susceptibility, and polymorphism in such gene families is a major risk. Genetic variations in phase I and/or phase II genes may alter the activity of the corresponding enzyme responsible for the bioactivation of xenobiotics; individuals with genetically impaired xenobiotic elimination functions will be at higher risk of disease susceptibility over their lifetime. Identification of the genes responsible for the elimination of xenobiotics and their variability is key for the identification of individuals at higher risk [12,33,34,35].

## 3. Evidence of Xeno-Estrogenicity

Since the advent of modern agriculture, pesticide use has become a primary means to enhance crop productivity. Amongst all the pesticides, OCPs became the first choice of farmers around the world after their introduction, attributed to their low cost and versatile effectiveness against a wide range of agricultural pests. Table 1 presents the current usage status, commercial name, and other relevant details related to pesticides. Organochlorine pesticides were banned in developed countries after their listing in the Stockholm Convention on Persistent Organic Pollutants (an international agreement aimed at the banning of harmful environmental pollutants), which is still used for the development and control of vector-borne diseases such as malaria. The overuse of OCPs has been rising alarmingly, and it is raising serious threats to both human health and the environment [36]. Every year, billions of pounds of OCPs are used for boosting agriculture productivity to fulfill the dietary needs of the world population. It is not surprising that high levels of these harmful chemicals are also detected in water and soil [37,38].

The hormone-like activity of these man-made chemicals was realized long after they were released into our environment. Krishnan et al. (1993) accidentally found unknown chemicals to be estrogenic because they disrupted experiments conducted in laboratories while studying the effects of natural estrogens. Later experiments confirmed that the disruption of the experiments was due to the plastic tubes used in the experiments possessing estrogenic activity [42]. In humans, indirect positive evidence of “pesticide exposure-related estrogenicity” was reported among farm workers. In 1949, workers occupationally exposed to dichlorodiphenyltrichloroethane (DDT) were found to have significantly lowered sperm counts and loss of fertility [43].

These chemicals are known to stay in the environment for a long period of time, which could be from a few years to several decades in different media; for instance, the most well-known pesticide, DDT, has a half-life of up to 30 years in soil and 3 to 6 years in the human body (up to 150 years in water) [44]. Therefore, long-term exposure may lead to considerable accumulation in adipose tissue due to the lipophilic nature of agricultural chemicals [45]. High levels of pesticides, including OCPs, have been reported in different samples from all over the world, including blood, adipose tissue, placental tissue, umbilical cord, colostrum, and even cord blood, etc. [6,7,8]. In a recent study, groundwater polluted with pesticides was found to increase susceptibility to estrogen-mediated cancers, in spite of the ban on these chemicals for decades [46]. Likewise, alarmingly high levels of these chemicals have also been reported from other regions of the world in recent studies [47,48,49]. Additionally, a Brazilian study from the year 2022 linked pesticide residue with an increased risk of cancer in one province [50].

These xenobiotics contaminate water resources and animals living in pesticide-contaminated water, leading to their accumulation in the animals’ bodies. The quantity of OCPs in their bodies can be several folds higher than that in the surrounding water. If these animals are consumed by other organisms, the OCPs will enter and accumulate in those animals’ bodies (bioaccumulation) [51]. Studies on the OCP levels in edible fishes found alarmingly high levels of these compounds [52,53,54], suggesting that these compounds are bioaccumulating in our bodies at an alarming rate, mainly due to the biomagnification of pesticides through seafood. These compounds are still found in our food and increasing the risk of human diseases despite the ban.

## 4. Confirmation of the Pesticide’s Xeno-Estrogenicity

Data from the observation of occupational exposure were not sufficient to understand the adverse effects of these OCPs. Nevertheless, animal- and cell-line-based studies unambiguously confirmed the xeno-estrogenic potential of heavily used and well-known pesticides [3,55,56].

Animal-based experiments first confirmed the biological response to DDT comparable to that for estrogens, such as increased uterine weight and vaginal epithelial cornification in lab animals, known as uterotrophic assay [57]. Later, uterotrophic assays of aldrin, dieldrin, endosulfan, and HCH confirmed that all these compounds were xeno-estrogenic in nature [55,56]. Furthermore, a cell-line-based study found evidence of the pesticides modulating estrogen-dependent genes as natural estrogens do [58].

The highest isoform of dichlorodiphenyltrichloroethane, the most well-known pesticide in the world, *p,p*′-DDT (more than 85% of *w*/*w*), was found to be estrogenic in animal and cell lines experiments [59]. Additionally, experiments proved that DDT metabolite binds to the estrogen receptor (ER), further consolidating its xeno-estrogenic activity [60].

Dieldrin was reported to cause various forms of cancer in at least seven strains of mice upon oral administration [61]. In one study, dieldrin was found to decrease the binding of natural estrogens to its receptor in female rats’ uterine tissue extracts. Additionally, in another animal-based study, the dieldrin treatment was found to competitively inhibit the binding of 17β-estradiol (the most potent form of estrogen) to the receptor in the rat uterus, indicating the strong similarities between these two compounds [62]. Dieldrin treatment at a concentration of 10 µM in MCF-7 cells produced a significant increase in proliferation. The proliferation induced due to dieldrin at this concentration was 54.89% that of estradiol, indicating that it is a strong xeno-estrogen [63]. Both isoforms of the pesticide endosulfan, α- and β-endosulfan, were reported to possess xeno-estrogenic potential [64]. Furthermore, atrophy was noticed in different testicular tissues of male rats fed meal containing 10 ppm concentration of endosulfan [65]. In another study, it was noted that endosulfan-induced atrophy relates to biochemical changes in testicular tissue that translate into a significant fall in sperm counts in the testicular epididymis region and reduced spermatid counts in intratesticular tissue [66]. Endosulfan-induced testicular atrophy has also been reported to induce infertility in males due to a dramatic decrease in sperm count after exposure [67].

Chemicals produced by humans and nature possess estrogenic activity. However, the molecular details or structure rendering a chemical/compound its xeno-estrogenic activity are not well known. However, the majority of environmental xeno-estrogens share a common structural motif either of phenol or structurally similar to phenol as observed in all the OCPs [68].

**Figure 1:**
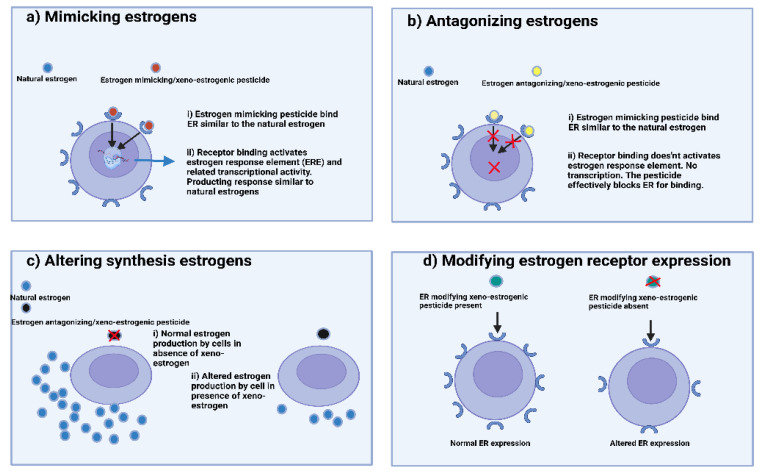
Different mechanisms of endocrine disruption by xeno-estrogenic pesticides: (**a**) mimicking estrogens, (**b**) antagonizing estrogens, (**c**) altering estrogen synthesis, and (**d**) modifying estrogen receptor expression.

The understanding of mechanisms of endocrine disruptors exerted by such chemicals is growing with time. Scientists proposed that by following these mechanisms, these environmental chemicals (including OCPs) are reported to lead to endocrine disruption (including xeno-estrogenicity) [69]. Figure 1 presents the different mechanisms of action of these compounds:

1. Mimicking the effect of endogenous steroidal hormones (androgens and estrogens).
2. Antagonizing steroidal hormones.
3. Altering the synthesis and metabolism of endogenous steroidal hormones.
4. Modifying hormone receptor expression in different tissues.

## 5. Association of Xeno-Estrogenic Pesticides with Endocrine-Related Cancer

Through human studies, the role of estrogens in female as well as male cancers has been well established. Breast and endometrial cancers in females are primarily estrogen-mediated [70]. In males, declining testosterone with age and rising estrogens are known to elevate prostate cancer risk with age [71].

Therefore, the risk of estrogen-mediated cancer has been analyzed in relation to exposure to OCPs by researchers. Risk of breast, endometrium, and ovary cancers in females and risk of prostate and testis cancers in males are estrogen-dependent to some extent [71]. Studies indicate a strong relation between exposure to estrogens as the principal risk factor in the development of these estrogen-mediated cancers [15,16,18,19,32,38,57,60,62,70].

### 5.1. Xeno-Estrogenic Pesticides and Female Cancer

The role of these xeno-estrogenic pesticides in elevated breast cancer risk has been most extensively studied compared to any other cancer in humans [11,72,73,74,75,76,77,78,79,80,81,82,83]. Over the past few decades, the incidence and mortality of breast cancer have increased worldwide by more than 33% [72,73]. Numerous studies have linked the contaminants we humans are unavoidably exposed to as the prime cause of higher breast cancer risk. Sufficient evidence points to the fact that breast cancer is strongly related to exposure to such contaminants, including DDT and other pesticides that act as estrogenic stimulants [40]. Significantly higher levels of pesticides such as DDT along with its persistent metabolites (especially DDE and DDD), heptachlor, dieldrin, and hexacyclohexane were detected in samples of patients with carcinoma of the breast, irrespective of any other factor when compared to controls [74].

Incidence of breast carcinoma is the world’s second highest, and it has the fifth highest mortality rate, while 36.8% of all incidences directly link to lifestyle and/or environmental factors in females over the age of 30 years. A significant association was found between the breast cancer epidemic and environmental contaminants exposure—especially DDT [11,75]. In one of the most significant studies linking OCPs with breast cancer, the decline in mortality rates observed in a decade (1976 to 1986) related significantly to decreases in specific OCPs (DDT and lindane) in colostrum [38]. Studies also suggest that removal of xeno-estrogens can prevent the incidence of and mortality due to breast cancer in females [76]. In prospective studies, exposure to OCPs was measured over a long time before diagnosis, and a statistically significant link was reported between breast cancer risk and DDT exposure. Similarly, two studies consistently linked the pesticide DDT with higher breast cancer risk when significant exposure occurred before teenage years (below the age of 14 years) [77,78]. These observations were further consolidated by a cohort study where it was found that above a certain cut-off concentration of dieldrin (>57.6 ng/g), morbidity and mortality amongst breast cancer patients significantly increased [78]. Dieldrin weakly induced both ER expression and cell proliferation in MCF-7 cell lines in a study, indicating dieldrin to be weakly estrogenic in females [82].

It is not clear how the estrogenic mechanism increases breast cancer risk. In a toxicoproteomic study amongst breast cancer patients, these OCPs were found to downregulate the expression of ER (a common event in a large number of breast cancer cases) by disrupting the relevant pathways [83]. In vitro studies also confirmed estrogen deregulation and increases in the concentration of cellular metabolites that activate a number of oncogenes [84,85]. In a recent study from China, OCPs were not only associated with breast cancer risk but were also found to elevate oxidative stress biomarkers in both serum ad urine [86].

Studies analyzing the role of xeno-estrogenic pesticides in endometrial cancer risk are few and rather inconclusive. Previous studies did not find any correlation between xeno-estrogenic OCP levels and endometrial cancer risk [87,88]. More human and animal experiment-based studies are needed to establish a link between these contaminants and endometrial cancer risk.

### 5.2. Xeno-Estrogenic Pesticides and Male Cancer

The role of xeno-estrogens in modifying the risk of male cancer and other related disorders is heavily debated and not well understood. In utero and early postnatal estrogen exposure is a significant contributory factor for testicular cancer risk in young men [89,90]. Studies from our lab have found a high level of these pesticides in mothers’ milk and umbilical cord, indicating continuous transfer of these pesticides during the early development of children [6,7,8].

Endocrine-disrupting chemicals were found to contribute to increased testicular cancer risk in some studies [91,92,93]. Later, this hypothesis was further consolidated when it was found that exposure to different xeno-estrogens in utero increases testicular cancer risk by fourfold in men (between the ages of 16 and 59) [64]. In an occupational study, xeno-estrogenic OCPs were found to increase testicular cancer risk among youngsters born to Norwegian farmers from 1952 to 1991, reporting a higher-than-expected testicular cancer incidence compared to controls [94]. In a nested case–control study, it was found that mothers of patients with testicular cancer had significantly higher DDT metabolites in their body during the lactational period [95]. A pooled study found 1.29 times higher testicular dysfunction risk, the main cause of testicular cancer, amongst the population living in regions having higher occupational and non-occupational exposure to OCPs compared to those in regions with limited or lower chances of exposure [96].

Due to the hormonal basis of prostate development, researchers have valid reasons to understand the potential relationship of xeno-estrogenic pesticides with prostate cancer risk [97]. The human prostate gland is the organ most affected by malignant neoplasm in elderly men. With the increase in life expectancy, the incidence of cancer has increased, affecting almost 90% of males over 80 years of age. The basis for this high incidence is not clear despite decades of exhaustive research [98]. In contrast to testicular cancer, direct connections between xeno-estrogenic pesticides and prostate cancer have not been established [90,99]. Animal- and cell-line-based studies are a better indicator of the effect of xeno-estrogenic pesticides on the functioning and transformation of prostate cancer. One study reported that endocrine-disrupting chemicals can transform and/or reprogram prostate stem cells and potentially elevate prostate cancer risk in experimental animals [100]. In an animal-based study, it was found that feeding regular small doses of xeno-estrogens to pregnant females led to a significantly increased prostate weight in adulthood in their pups; increased prostate weight is one of the hallmarks of prostatic disorders, including both prostate cancer and benign prostatic hyperplasia (BPH) [101]. The most compelling proof for a link between xeno-estrogenic OCP exposure and prostate cancer comes from studies from our laboratory. We found that the levels of some of these pesticides were significantly higher amongst patients with higher-stage and more aggressive prostate cancer, indicating the development of an aggressive form of cancer. In addition, these pesticides influence prostate weight as well amongst BPH patients [102,103]. Occupational pesticide exposure is regarded as an established factor elevating prostate cancer risk [104,105,106]. Furthermore, numerous epidemiological studies reported a positive relation between (non-occupational) pesticide exposure and the risk of prostate cancer [106,107,108]. In relatively recent Asian studies, DDT and endosulfan were found to increase prostate cancer risk. This hints that in spite of the partial ban on these two compounds, they may still be increasing the risk [109,110]. However, more studies are needed to understand if there is a relation before drawing any conclusion.

## 6. Conclusions

**Figure 2:**
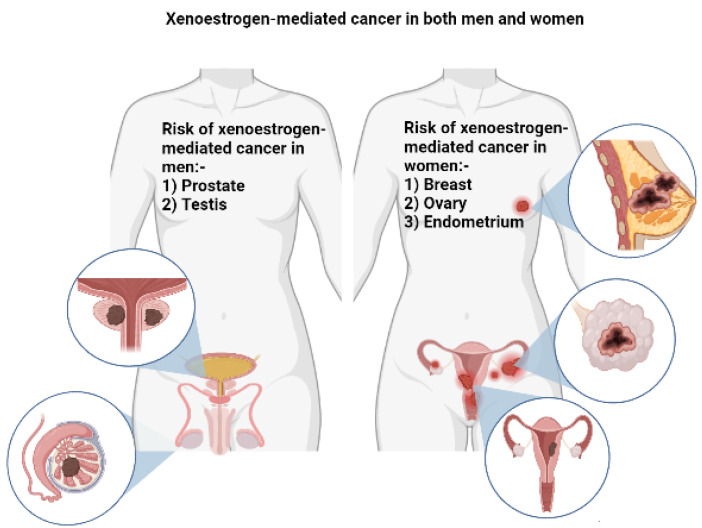
Risk of various estrogen-mediated cancers with xeno-estrogenic pesticide exposure.

The association of pesticides, including OCPs, with cancer risk is well established. However, some of the mechanisms, such as xeno-estrogenicity, that increase the risk of carcinogenic progression are not clearly understood. Accumulating evidence is consolidating the role of xeno-estrogenic OCPs in the risk of not only cancers but also a plethora of other human disorders. These chemicals, despite their apparent adverse health effects, are still used in some countries. Although the risk of some cancers such as breast cancer is clearly modified with exposure to the discussed chemicals, the link with the risk of other estrogen-mediated cancers, especially in males, is not clearly understood and needs to be studied more. Figure 2 represents the risk of different cancers reported to be associated with exposure to xeno-estrogenic pesticides. In recent decades, breast cancer in females and prostate cancer in males have become the top causes of morbidity and mortality, and both are estrogen-mediated cancers. More studies are needed to find out how much this increased incidence can be attributed to such harmful environmental factors. To the best of our knowledge, this is the only study that focuses on all-gender estrogen-mediated cancer risk modification by xeno-estrogenic OCPs. These chemicals must be completely phased out and replaced with less toxic and affordable alternatives that have negligible adverse health effects on mammalian systems.

## Figures and Tables

**Table 1:** Current use status and year listing of commercial pesticide brands around the world.

Name of Pesticide	Commercial Name	Ban Status	Current Status	Year of Listing in Stockholm Convention on Persistent Organic Pollutants	Used for the Crops	References
Dieldrin	Hortico, Dieldrin Dust Mustex 25%, Shell Dieldrex, Yates Garden Dust	Complete	No manufacturing	2001	Corn, cotton, citrus cabbage, legumes	[39]
DDT	Cezarex, Anofex, Clorophenothane, Dicophane, Dinocide, Gesarol, Guesapon, Guesarol, Gyron, Ixodex, Neocid, Neocidol, Zerdane	Partial	For malaria control programs (countries of Africa and Asia)	2001	Amaranth, cabbage, lettuce, pumpkin, spinach	[40]
Endosulfan	Afidan, Beosit, Endocel, Hildan Cyclodan, Devisulfan, Endocide, Endosol, FMC 5462, Hex-asulfan, Hoe 2671, Insectophene, Malix, Thiodan, Thimul, Thifor, Thionex	Regulated	For certain crop–pest complexes	2011	Spinach, cauliflower, potato, brinjal, tomato, okra	[41]
Aldrin	Aldrec, Aldrex, Drinox, Octalene, Seedrin, Compound 118	Complete	No manufacturing	2007	Corn, cotton, citrus, cabbage, legumes	[39]
HCH	Forlin, Etan 3G, Gamaphex, Isotox, GermatePlus, Gamma-Mean400, Lindagram	Complete	No manufacturing	2008	Amaranth, cabbage, lettuce, pumpkin, spinach	[40]

## Data Availability

The data used to support the findings of the study are included within the article.
